# The predictive value of intraoperative visual evoked potential monitoring for postoperative visual outcomes following extended endoscopic endonasal resection of recurrent craniopharyngiomas

**DOI:** 10.1186/s41016-026-00425-x

**Published:** 2026-02-27

**Authors:** Xiaorong Tao, Ke Li, Xiaocui Yang, Jiajia Liu, Jun Yang, Jiawei Shi, Yingzhun Liang, Songbai Gui, Chuzhong Li, Xing Fan, Hui Qiao

**Affiliations:** 1 Department of Neurophysiology, Beijing Neurosurgical Institute, Capital Medical University, Beijing, China; 2 Department of Neurosurgery, Beijing Tiantan Hospital, Capital Medical University, Beijing, China

**Keywords:** Recurrent craniopharyngioma, Extended endoscopic endonasal approach, Intraoperative monitoring, Visual evoked potential

## Abstract

**Background:**

Recurrent craniopharyngiomas pose high risks of postoperative visual dysfunction (POVD) during surgery. The current study aimed to explore the application value of intraoperative visual evoked potential (VEP) monitoring during the extended endonasal endoscopic approach (EEEA) for recurrent craniopharyngiomas.

**Methods:**

A total of 42 patients with recurrent craniopharyngiomas undergoing EEEA with VEP monitoring were analyzed. The amplitude reduction ratios of N75-P100 and P100-N145 were calculated, and their predictive values for POVD were evaluated using group comparisons, receiver operating characteristic (ROC) curve analysis, and binary logistic regression analysis.

**Results:**

POVD was observed in 8 eyes (8/84, 9.52%) from 7 patients (7/42, 16.67%). Eyes with POVD exhibited significantly greater N75-P100 and P100-N145 amplitude reduction ratios than those without (p < 0.001 and p = 0.002, respectively). The threshold values of the two ratios for predicting POVD were 36.59% (AUC 0.862, p < 0.001) and 36.65% (AUC 0.791, p=0.007), respectively. Multivariate analysis identified that abnormal N75-P100 change was the sole independent predictor of POVD (Odds ratio 9.257, 95% Confidence interval 1.124-76.263; p = 0.039).

**Conclusions:**

Intraoperative VEP monitoring was particularly recommended for patients undergoing EEEA for recurrent craniopharyngiomas. A one-third reduction in N75-P100 amplitude was proposed as an early warning criterion for VEP monitoring in this patient population.

## Background

Craniopharyngiomas, including adamantinomatous and papillary craniopharyngiomas, are rare developmental tumors arising in the sellar region [1, 2]. Despite being categorized as benign according to the latest World Health Organization classification, about 50 percent of them show invasive behavior and are prone to recurrence [2, 3]. Due to their anatomical proximity to critical structures such as the pituitary, hypothalamus, optic nerves, and optic chiasm, achieving gross-total resection, which is the gold standard for craniopharyngioma management, is often technically challenging [4]. In consequence, some neurosurgeons have increasingly regarded subtotal resection followed by adjuvant radiotherapy as a preferred treatment strategy [5, 6]. The advent of the extended endonasal endoscopic approach (EEEA) has transformed the aforementioned therapeutic paradigm, offering improved rates of gross-total resection and reduced surgical complication risks [7, 8].

Postoperative visual dysfunction (POVD) represents a major complication following craniopharyngioma resection, with EEEA also carrying this risk [9, 10]. In addition to incomplete tumor removal, surgical injury to the optic nerves or chiasm is also a critical contributor to the development of POVD. To minimize the risk of iatrogenic injury, the application of intraoperative visual evoked potential (VEP) monitoring provides a promising and effective strategy [11, 12]. Intraoperative VEP monitoring can provide real-time insights into the functional integrity of the visual pathway. This enables neurosurgeons to quickly detect and respond to potential damage to the optic nerves or chiasm, thus protecting them from irreversible severe injury.

Since the late 2010s, our team has been utilizing VEP monitoring in patients undergoing EEEA for craniopharyngiomas. Through systematic observation and analysis, we have demonstrated the application value of VEP monitoring in this context and have also proposed potential early warning criteria for its use [13-15]. Compared with primary craniopharyngiomas, recurrent craniopharyngiomas present greater surgical challenges and carry a higher risk of complications [16]. This highlights the importance of VEP monitoring for these cases. Regarding this issue, our preliminary findings have indicated that VEP monitoring remains effective during EEEA for recurrent craniopharyngiomas [17]. However, it remains unclear whether the early warning criteria for VEP monitoring during recurrent craniopharyngioma surgeries are the same as those for primary tumors. Given the distinct pathological and anatomical features of recurrent tumors, unique thresholds for VEP changes may be required for accurate early warning and the prediction of visual outcomes. In the current study, we investigated the correlation between intraoperative VEP amplitude changes and POVD, aiming to establish a foundation for developing personalized monitoring criteria to optimize visual pathway protection during surgery for recurrent cases.

## Methods

### Study cohort

This study included 42 consecutive patients who underwent EEEA for recurrent craniopharyngiomas between July 2019 and August 2022 at Beijing Tiantan Hospital, with intraoperative VEP monitoring performed during all procedures. The inclusion criteria were as follows: 1) pathologically diagnosed as recurrent craniopharyngioma, 2) underwent surgical resection via EEEA, 3) absence of severe preoperative visual impairments; 4) successful acquisition of VEP monitoring data throughout the surgery, defined by stable baseline waveforms and identifiable response changes, and 5) available demographic and clinical data. This study was approved by the Ethics Committee of Beijing Tiantan Hospital. Written informed consent was obtained from all patients or their legal representatives prior to surgery.

### Anesthesia

Total intravenous anesthesia was induced with propofol (2–2.5 mg/kg) and sufentanil (0.3–0.4 μg/kg), and maintained via continuous infusion of propofol (4–12 mg/kg/h) and remifentanil (0.05–0.2 μg/kg/min). Bispectral index values were maintained within the target range of 40 to 60. Basic vital signs were continuously monitored throughout the surgical procedure.

### Intraoperative neurophysiological monitoring

VEP and electroretinogram (ERG) monitoring were obtained in all patients following the previously described protocol [15]. Visual stimulation was delivered using a light-emitting diode (LED) stimulator (Nim-Eclipse System, Medtronic Xomed Inc., Jacksonville, FL, USA). The brightness of the LED was set to 500-10,000 Lx, and the duration of each stimulus was 84 ms. Goggles were placed over both closed eyelids. VEP was recorded using corkscrew electrodes positioned bilaterally at the O1, O2, and Oz sites, as defined by the International 10–20 system. The recording parameters were set as follows: a band-pass filter of 1–100 Hz, signal averages<50, and an analysis time window of 300 ms. The ERG was recorded to confirm retinal activity after stimulation, with electrode placement positioned 2 cm lateral to the bilateral external canthi.

Baseline VEP was acquired following dura mater incision. VEP waveforms were recorded at 2-minute intervals during optic canal decompression and tumor resection. Early warning would be given when an over 50% reduction of N75-P100 or P100-N145 amplitude was observed, excluding anesthetic and physiological effects. N75, P100, and N145 are three wave peaks of the VEP waveform with different latencies, with “N” and “P” representing negative (upward) and positive (downward) polarity, respectively. The VEP amplitude reduction ratio was calculated via the following formula: (baseline amplitude - amplitude recorded at the end of surgery)/baseline amplitude × 100%. Negative values were recorded as zero.

### Visual Assessment and follow-up

Best-corrected visual acuity was measured preoperatively and at least 6 months postoperatively for all patients. Assessments were conducted by certified and experienced optometrists using a standardized logarithmic visual acuity chart at a distance of 5 meters under controlled lighting conditions. A decrease of at least one line on the chart was defined as POVD.

### Statistical analysis

The IBM SPSS Statistics software (Version 26.0, IBM Corp., Armonk, New York, USA) and GraphPad Prism software (Version 10.1.2, GraphPad Software Inc., San Diego, California, USA) were utilized for data management and statistical analysis. Continuous variables were presented as means accompanied by corresponding standard deviations, and categorical variables were characterized by frequencies or proportions. Group comparisons were performed using the Chi-square test, Fisher’s exact test, Student’s t-test, or Mann-Whitney U-test, depending on their relevance. Subsequently, receiver operating characteristic (ROC) curve analysis was employed to determine the threshold values of the N75-P100 or P100-N145 amplitude reduction ratios for predicting POVD, and the area under the curve (AUC) was calculated to assess its predictive accuracy. A p-value < 0.05 was considered statistically significant. A p-value less than 0.05 was considered statistically significant. At last, univariate and multivariate binary logistic regression analyses were conducted to further assess the predictive value of the N75-P100 and P100-N145 amplitude reduction ratios for POVD if necessary. The effects were presented as odds ratios (ORs) with their corresponding 95% confidence intervals (CIs).

## Results

### Patient characteristics

The study cohort comprised 17 male and 25 female participants, with a mean age of 44.07 ± 13.14 years (range, 22–67 years). Of the total cases, 29 (69.0%) were histopathologically diagnosed as adamantinomatous craniopharyngiomas, while 13 (31.0%) were classified as papillary craniopharyngiomas. The mean duration of preoperative visual impairment was 3.48 ± 4.60 months (range, 0–18 months). The mean recurrence time was 4.58 ± 4.77 years (range, 0.25–21.00 years).

### The correlation between the VEP amplitude reduction ratio and POVD

**Fig. 1:**
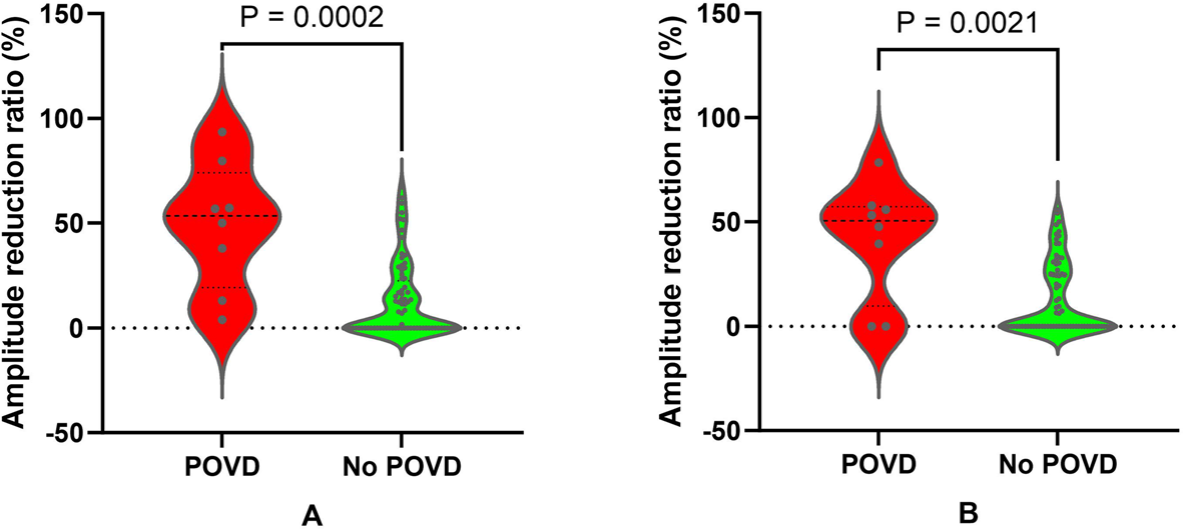
Comparison of VEP amplitude reduction ratios between eyes with and without POVD. A: N75-P100 amplitude reduction was significantly greater in POVD eyes (48.99% ± 30.43%) than in non-POVD eyes (p<0.001, Mann-Whitney U-test). B: P100-N145 amplitude reduction was also higher in POVD eyes versus non-POVD eyes (p=0.002, Mann-Whitney U-test)

Reproducible and reliable VEP waveforms were recorded from all 84 eyes of 42 patients. POVD was identified in 8 eyes (8/84, 9.52%) from 7 patients (7/42, 16.67%). No significant differences were observed in the aforementioned demographic characteristics between patients with POVD and those without (p >0.05 for all). Eyes with POVD exhibited significantly greater N75-P100 (48.99% ± 30.43% vs. 13.02% ± 18.00%, p < 0.001, Mann-Whitney U-test; Fig. 1A) and P100-N145 (41.49% ± 27.89% vs. 12.92% ± 17.19%, p = 0.002, Mann-Whitney U-test; Fig. 1B) amplitude reduction ratios compared to those without.

### The threshold values of the N75-P100 and P100-N145 amplitude reduction ratios for predicting POVD

**Fig. 2:**
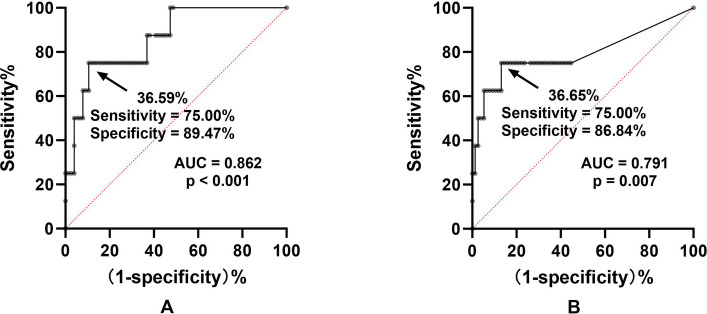
Receiver operating characteristic curves for VEP amplitude reduction ratios predicting POVD. A: N75-P100 amplitude reduction ratio predicted POVD with a threshold of 36.59%. B: P100-N145 amplitude reduction ratio predicted POVD with a threshold of 36.65%. AUC, area under the curve

ROC curve analysis was conducted to determine the predictive cutoff value of the N75-P100 amplitude reduction ratio for POVD. The results are illustrated in Fig. 2. The threshold value of the N75-P100 amplitude reduction ratio for predicting POVD was found to be 36.59%, with an AUC of 0.862 and a p-value of less than 0.001 (Fig. 2A). The threshold value of the P100-N145 amplitude reduction ratio was 36.65% (AUC 0.791, p=0.007, Fig. 2B).

### The predictive value and independence of abnormal N75-P100 and P100-N145 changes

Finally, univariate and multivariate binary logistic regression analyses were performed to assess the predictive value and independence of abnormal changes in N75-P100 and P100-N145 for POVD. If an amplitude reduction ratio for N75-P100 or P100-N145 exceeds the previously established threshold, it is classified as an abnormal change. Both abnormal N75-P100 and P100-N145 changes showed significant associations with POVD in univariate analysis (p < 0.001 and p = 0.001, respectively). However, in multivariate analysis, only the abnormal N75-P100 change remained as an independent predictor of POVD (OR 9.257, 95% CI 1.124–76.263; p = 0.039). A Pearson correlation analysis was carried out to assess potential collinearity between the two ratios. The results revealed a moderate positive linear relationship, with a correlation coefficient of 0.685 (p < 0.001).

## Discussion

The current study evaluated the predictive value of VEP monitoring during EEEA for postoperative visual outcomes in patients with recurrent craniopharyngiomas. The N75-P100 amplitude reduction ratio was identified to be an independent predictor for POVD. The findings could provide meaningful insights for refining intraoperative warning criteria in this patient population and contribute to improved management of patient expectations.

As early as 1973, Wright et al. first attempted to perform VEP monitoring in intraorbital surgery [18]. However, the application of this technique was limited due to unreliable waveform recordings, primarily as a result of the technological constraints of that time. In recent years, substantial advancements in VEP monitoring have emerged across various domains, including stimulation methods, recording techniques, and data analysis approaches. These improvements have significantly enhanced the reliability and clinical effectiveness of VEP monitoring. For instance, the introduction of high-brightness LED stimulators has improved both the stability and controllability of the stimulation, ensuring that the retina receives effective stimulation and thereby elevating the quality of VEP signals [19]. Furthermore, the optimization of the anesthesia protocol, primarily the introduction of total intravenous anesthesia, has also significantly improved the outcomes of VEP monitoring [20, 21]. To date, intraoperative VEP monitoring has developed into a safe, reliable, and progressively effective method for preserving visual function during surgical procedures [22].

The EEEA for craniopharyngioma was the first surgical procedure in which we implemented VEP monitoring, and we have systematically validated the clinical utility of VEP monitoring in this approach through a series of studies [13, 17]. However, refining and defining a precise early warning criterion for VEP monitoring remains an ongoing objective for our team. In 2021, we proposed that a latency prolongation of approximately 10% could serve as an early warning criterion for VEP monitoring during EEEA for craniopharyngioma [14]. In 2024, we recommended that both reductions in N75-P100 and P100-N145 amplitudes should be given due attention, with warning thresholds set at 50% and 40% decreases, respectively [15]. In the current study on recurrent craniopharyngiomas, we found that only the change in N75-P100 amplitude warranted intraoperative attention, whereas the P100-N145 amplitude lost its predictive significance. Furthermore, the early warning criterion for the N75-P100 amplitude reduction ratio during EEEA in recurrent craniopharyngiomas is 36.59%.

The aforementioned series of studies collectively highlights a critical issue. Different early warning criteria are required for the intraoperative monitoring of primary and recurrent tumors, even when the tumor type and surgical approach are identical. The threshold value of N75-P100 amplitude reduction during EEEA for recurrent craniopharyngiomas in predicting POVD (36.59%) is markedly lower than the 51.76% observed in primary tumors [15]. This observation reflects the greater complexity of surgery for recurrent tumors, which are often closely attached to surrounding neurovascular structures, including the optic nerve and chiasm, making it particularly challenging to preserve visual function. Overall, VEP monitoring is more necessary for the resection of recurrent craniopharyngiomas. Considering clinical feasibility, we recommended a one-third reduction in N75-P100 amplitude as the early warning criterion for VEP monitoring during EEEA for recurrent craniopharyngiomas.

Another point that needs to be emphasized is that during VEP monitoring for recurrent craniopharyngiomas, the P100-N145 amplitude changes may not require routine intraoperative attention. It is important to clarify whether this finding conflicts with our previous results in primary craniopharyngiomas [15]. In addition to the unresolved mechanistic complexities of VEP, we speculate that the following factors may collectively contribute to the diminished predictive value of the P100-N145 component in this study. First, according to our prior study, N75-P100 demonstrates a superior predictive value for postoperative visual dysfunction (POVD) compared to P100-N145. Second, as distinct components of the VEP waveform, N75-P100 and P100-N145 inherently share a moderate degree of collinearity. Third, the sample size of the current study was relatively small (42 patients with 8 POVD eyes). Notably, our re-evaluation of primary craniopharyngioma data showed that although the collinearity between N75-P100 and P100-N145 was similar to that observed in the current study, it did not compromise the independent predictive value of P100-N145.

It is important to acknowledge that our analysis focused on the amplitude reduction from baseline to the end of surgery. This approach, while providing a clear and reproducible metric, does not capture the potential prognostic value of transient amplitude changes that occur during surgical manipulation. Extensive literature indicates that VEP amplitude may decrease temporarily during critical steps but recover by the end of the procedure, a phenomenon that could mask intraoperative injury [11]. Future studies incorporating high-frequency, time-locked VEP recordings throughout key surgical maneuvers are warranted to explore the predictive power of these dynamic changes and potentially establish even earlier warning signs.

In summary, the apparent discrepancy between the current study and our prior work does not represent a conflict but rather illustrates the progressive refinement of our understanding of VEP monitoring within the context of evolving clinical practice. This progression aligns with the principle of individualized neurophysiological monitoring, whereby monitoring criteria are specifically adapted to each patient subgroup’s pathological and surgical characteristics, thereby improving the precision and effectiveness of visual function preservation during craniopharyngioma resection.

Several limitations of this study should be acknowledged. First, the relatively small sample size and limited number of POVD events may reduce the statistical power of the results and the generalizability of the proposed threshold. Second, the retrospective design inherently introduces potential selection bias. Third, the visual assessment was based on visual acuity without systematic visual field analysis (perimetry), which is a more sensitive measure for chiasmal syndromes commonly associated with craniopharyngiomas; this was primarily due to logistical constraints in obtaining complete perimetry data during follow-up. Fourth, preoperative VEP assessment was not routinely conducted, which could have provided a more personalized baseline. Finally, as noted in the discussion, our analysis was limited to comparing baseline and end-of-surgery amplitudes, omitting the relationship between reversible intraoperative amplitude fluctuations and POVD. Given these limitations, future prospective, large-sample studies incorporating preoperative VEP, standardized visual field testing, and dynamic intraoperative amplitude analysis are warranted to validate our findings and refine individualized monitoring protocols.

## Conclusions

In the current study, we evaluated the clinical value of VEP monitoring during EEEA for recurrent craniopharyngiomas. Intraoperative N75-P100 amplitude reduction was identified as a robust and independent predictor of POVD. Furthermore, we proposed a one-third reduction in N75-P100 amplitude as an early warning criterion for VEP monitoring in this high-risk and technically challenging patient population. The results could contribute to refining intraoperative VEP monitoring protocols for recurrent craniopharyngiomas and provide more substantial evidence supporting the exploration of individualized intraoperative monitoring.

## Data Availability

The datasets used and/or analysed during the current study are available from the corresponding author on reasonable request.

## Abbreviations

EEEA: Extended Endoscopic Endonasal Approach
VEP: Visual Evoked Potential
POVD: Postoperative Visual Dysfunction
ROC: Receiver Operating Characteristic
AUC: Area Under the Curve
ERG: Electroretinogram
LED: Light-Emitting Diode
OR: Odds Ratio
CI: Confidence Interval
